# CNS Remyelination and the Innate Immune System

**DOI:** 10.3389/fcell.2016.00038

**Published:** 2016-05-03

**Authors:** Christopher E. McMurran, Clare A. Jones, Denise C. Fitzgerald, Robin J. M. Franklin

**Affiliations:** ^1^Wellcome Trust-Medical Research Council Cambridge Stem Cell Institute, University of CambridgeCambridge, UK; ^2^MedImmuneCambridge, UK; ^3^Centre for Infection and Immunity, School of Medicine, Dentistry and Biomedical Science, Queens University BelfastBelfast, UK

**Keywords:** remyelination, inflammation, innate immune system, microglia, macrophage

## Abstract

A misguided inflammatory response is frequently implicated in myelin damage. Particularly prominent among myelin diseases, multiple sclerosis (MS) is an autoimmune condition, with immune–mediated damage central to its etiology. Nevertheless, a robust inflammatory response is also essential for the efficient regeneration of myelin sheaths after such injury. Here, we discuss the functions of inflammation that promote remyelination, and how these have been experimentally disentangled from the pathological facets of the immune response. We focus on the contributions that resident microglia and monocyte-derived macrophages make to remyelination and compare the roles of these two populations of innate immune cells. Finally, the current literature is framed in the context of developing therapies that manipulate the innate immune response to promote remyelination in clinical myelin disease.

## Remyelination: regeneration in the CNS

The mammalian central nervous system (CNS) is often considered an archetypal example of a tissue with poor regenerative potential. This is exemplified by clinical conditions such as spinal cord injury, stroke, and Alzheimer's disease, where prognosis is poor due to limited regeneration of neurons and axons (Tanaka and Ferretti, 2009). Myelin sheaths, however, are an important exception to this dogma, being a component of the CNS that *can* regenerate robustly with good functional outcome; a process termed remyelination (Franklin and Goldman, 2015).

Myelin sheaths are made up of layers of lipid-rich dielectric membrane wrapped around axons to which they provide electrical insulation and trophic support (Nave and Trapp, 2008). This membrane is produced by specialized glial cells: oligodendrocytes in the CNS, or Schwann cells in the peripheral nervous system (PNS). The loss of myelin sheaths with preservation of the underlying axon is known as demyelination. This is sometimes referred to as primary demyelination to distinguish it from secondary demyelination, where myelin loss occurs as a consequence of axonal loss. This latter process is more accurately referred to as Wallerian degeneration, and we regard the use of the term demyelination in this situation as confusing and misleading.

Remyelination involves the reinvestment of new myelin sheaths around intact axons from which they have been lost (i.e., demyelination; Franklin and Goldman, 2015). This process is performed by newly generated oligodendrocytes that derive from a pool of oligodendrocyte progenitor cells (OPCs) following a demyelinating insult. OPCs are present throughout both gray and white matter in the CNS, and have “stem cell-like” properties such as multipotency and self-renewal (Franklin and ffrench-Constant, 2008). In response to demyelination, OPCs proliferate and migrate to the lesion site (Di Bello et al., 1999; Crawford et al., 2014) where they differentiate to mature oligodendrocytes or Schwann cells, extending processes to remyelinate denuded axons (Zawadzka et al., 2010). Consequently, saltatory conduction is restored (Smith et al., 1979) and axons are generally protected from further degeneration (Irvine and Blakemore, 2008). In some paradigms, whilst axons are not fully protected, their degeneration is substantially delayed with motor deficits not re-appearing until much later timepoints (Manrique-Hoyos et al., 2012).

Whilst originally characterized in animal models (Bunge et al., 1961), remyelination is also seen in human patients with MS (Prineas and Connell, 1979). Amongst MS lesions there is an associated between remyelination and preservation of axons (Kornek et al., 2000), although it is in practice difficult to asses whether remyelination occurs because axons have survived, or the axons have survived because they are remyelinated. Whilst extensive in some cases, remyelination efficiency falls as the disease progresses, so it is usually insufficient to prevent a patient's neurological decline as damage gradually accumulates (Goldschmidt et al., 2009; Franklin et al., 2012).

Crucially, regenerative processes become less efficient with increasing age, and remyelination is no exception (Shields et al., 1999). This tenet of regenerative medicine is particularly relevant in a chronic disease such as MS, which spans several decades (Franklin, 2002). Aging brings about intrinsic changes in OPCs (Shen et al., 2008) and their environmental signals (Zhao et al., 2006), both of which negatively impact remyelination. Because of this age-related decline, many key findings have come from comparing remyelination or clinical outcome in young and old animals (Hinks and Franklin, 2000; Zhao et al., 2006; Shen et al., 2008) or human cases (Confavreux and Vukusic, 2006). More interventional approaches have manipulated these systems to identify pathways crucial for efficient remyelination in young animals (Kotter et al., 2001; Lampron et al., 2015; Natrajan et al., 2015) or that can rejuvenate remyelination in older animals (Ruckh et al., 2012; Miron et al., 2013).

When remyelination fails, the limiting step is most commonly OPC differentiation, a term encompassing the establishment of axonal contact, activation of myelin synthesis pathways and the wrapping and compaction of the newly generated sheath (Franklin and ffrench-Constant, 2008). In humans, this is evidenced by an abundance of undifferentiated oligodendrocyte lineage cells in many chronic MS lesions, which fail to remyelinate (Wolswijk, 1998; Kuhlmann et al., 2008). Thus, there is much clinical need for therapies to enhance OPC differentiation and endogenous remyelination. One avenue for this is to target the innate immune system.

## Innate immune cells of the CNS

The immune system is the network of cellular and molecular elements that protect an organism from disease. In vertebrates, it can broadly be divided into the innate and the adaptive immune systems, though these two branches communicate extensively and various components, such as innate lymphoid cells, share features of both. In general, the innate immune system responds rapidly and relatively non-specifically to infection or damage, whilst the adaptive immune system mounts a slower, long-lasting response to specific targets. Inflammation describes an immune-mediated response to stimuli that are perceived as harmful, such as invading pathogens or tissue damage.

Like other tissues, the CNS has its own battalion of resident tissue macrophages, called microglia. These are innate immune cells that, in the healthy brain, serve physiological functions in synaptic plasticity (Paolicelli et al., 2011) and clearance of debris (Ferrer et al., 1990). Meanwhile, microglia continuously survey their microenvironment for stimuli that might indicate injury or infection, and are ready to respond to this by entering an activated state characterized by cytokine secretion, phagocytosis and, on occasions, direct cytotoxicity (Nimmerjahn et al., 2005).

After such a stimulus, microglia may be supplemented by a second population of macrophages from the periphery. These differentiate from blood monocytes that have infiltrated the CNS in response to tissue damage, and thus originate in the bone marrow. Key differences exist between resident microglia and these monocyte-derived macrophages, including their disparate developmental origins (Ginhoux et al., 2010), their transcriptomic signatures (Hickman et al., 2013) and capacity for local self-renewal and expansion (Ajami et al., 2007). However, in a demyelinated lesion, both cell types show high levels of activation and only recently have successful attempts been made to phenotypically and functionally distinguish between them, as will be discussed.

Besides microglia and monocyte-derived macrophages, an array of other innate immune cells can be found in a demyelinating lesion. Whilst neutrophils (Liu et al., 2010), mast cells (Letourneau et al., 2003), and dendritic cells (Karni et al., 2006) can contribute to demyelination, with a disputed role of natural killer cells in promoting/limiting damage (Maghazachi, 2012), little evidence exists to support a substantial contribution of these cell types to remyelination (Mayo et al., 2012). Thus, in our discussion of CNS remyelination and the innate immune system, our focus is primarily on microglia and infiltrating macrophages.

## Innate immune cells and myelin disease

In MS, aberrant activity of the innate immune system can contribute to myelin damage. The driver of this is a defective adaptive immune response involving autoreactive T cells, but through innate-adaptive cross talk, microglia and monocyte-derived macrophages can be recruited and mediate a substantial part of the damage (Compston and Coles, 2002). Based on this etiology, clinical therapies for MS tend to focus on inhibiting the adaptive immune system. Examples include the monoclonal antibodies natalizumab, which impairs T cell trafficking into the CNS (Polman et al., 2006), and rituximab, which targets CD20 to deplete autoantibody-producing B cells (Castillo-Trivino et al., 2013).

A commonly used range of models for MS is experimental autoimmune encephalomyelitis (EAE), in which an inoculated animal develops autoreactive T-cells targeting myelin. This is a useful paradigm for modeling many of the immunogenic features of MS (Robinson et al., 2014). However, the complex inflammatory pathogenesis of these models can mask beneficial regenerative effects of the innate immune system that may occur concurrently with negative, disease-contributing innate cell functions. As such, delineating specific regenerative functions of innate cells and signals in EAE is challenging. In contrast, toxin-induced models of demyelination, such as focal lysolecithin injection, have more discrete phases of demyelination and remyelination. As the myelin damage is initiated directly by the toxin, we can generally assume that immune cell activity during the remyelination phase is a *response* to myelin damage, rather than an on-going cause. We will see how such a response can indeed contribute to the process of remyelination.

## The critical role of the innate immune system

Making use of these toxin-induced demyelination models, a beneficial role of the innate immune system in remyelination was demonstrated in rats depleted of circulating monocytes (Kotter et al., 2001). This was achieved by systemic treatment with clodronate liposomes, which are toxic to cells that phagocytose them. Treated animals had fewer inflammatory cells in the lesion suggesting a muted innate immune response to tissue damage and, interestingly, a lower proportion of their axons were remyelinated post-lesion.

Conversely, by promoting inflammation it is possible to increase the efficiency of myelin formation. This was shown by using transplanted OPCs to myelinate retinal ganglion cell axons, which are normally unmyelinated prior to where they form the optic nerve (Setzu et al., 2006). Administration of the Toll-Like Receptor 2 agonist zymosan greatly increased the number of activated macrophages and the degree of myelination by the transplanted OPCs. Thus, the link between an inflammatory response and remyelination has been shown in both “loss-of-function” and “gain of function” experiments.

A beneficial role of the innate immune system in remyelination is less clear-cut in models where inflammation also contributes to the damage. In EAE, similar to MS, both microglia and infiltrating monocytes are implicated in demyelination (Fife et al., 2000; Heppner et al., 2005; Ajami et al., 2011). However, evidence also suggests that subsets of these cells can play beneficial roles in limiting damage and promoting remyelination. Macrophages can modulate their phenotypes in response to extracellular cues (Edwards et al., 2006), often termed “M1” (pro-inflammatory) or “M2” (anti-inflammatory) polarization for convenience, though in reality representing a spectrum with a broad degree of plasticity (Murray et al., 2014). A shift toward the anti-inflammatory M2 phenotype is associated with a milder clinical picture in EAE (Mikita et al., 2011), and administration of M2-polarized monocytes to EAE animals can enhance differentiation of oligodendrocytes and improve symptoms (Butovsky et al., 2006). This is consistent with a crucial shift from pro- to anti-inflammatory phenotypes in remyelination of toxin-induced lesions (Miron et al., 2013). Myeloid-derived suppressor cells are a subpopulation of innate immune cells that typically express M2 markers and limit damage in EAE at peaks of disability, in part by promoting apoptosis of T lymphocytes (Moliné-Velázquez et al., 2011).

Thus, even on a backdrop of inflammatory damage, cells of the innate immune system can play beneficial roles in outcome and may contribute to remyelination. This is important for the application of findings from toxin-induced models to the immune-mediated pathology of MS. Indeed, post-mortem data from MS patients, shows a correlation between the density of macrophages and the density of OPCs within a lesion (Wolswijk, 2002). MS lesions can be classified by their histopathology: active plaques, which have on-going inflammation, show the highest levels of OPC recruitment and remyelination, whilst chronic active and chronic inactive plaques show little inflammation and remyelination is rare (Clemente et al., 2013). Lesions that remyelinate successfully become shadow plaques, in which axons are relatively preserved. Reactivation of inflammation in chronic plaques may be a strategy to overcome their remyelination failure (Franklin and Goldman, 2015).

The decline of remyelination efficiency with age also occurs in part at the level of the innate immune system. This was demonstrated using a heterochronic parabiosis system, in which mice of different ages are connected via their circulatory systems (Ruckh et al., 2012). Remyelination of a toxin-induced lesion is rejuvenated in old mice that are exposed to the systemic environment of a younger mouse. The effect was abrogated in mice that lacked CCR2, a receptor necessary for monocyte entry into the CNS, suggesting that young macrophages are able to stimulate old OPCs to differentiate more efficiently. A timely shift from pro- to anti-inflammatory innate immune cell phenotypes is important for efficient remyelination (Miron et al., 2013), a phenomenon also seen in regeneration of other tissues, including skin (Deonarine et al., 2007). This shift is diminished with aging, but rejuvenated by heterochronic parabiosis (Miron et al., 2013), and appears to be instrumental in the rejuvenation of remyelination.

The positive relationship between remyelination and inflammation is strikingly similar to results seen across a broad range of regeneration paradigms. Healing of other murine tissues such as skin (Mirza et al., 2009) is impaired when macrophages are subject to selective genetic ablation. The famously extensive regrowth of salamander limbs after amputation is also diminished when macrophages are depleted by a toxic liposome treatment (Godwin et al., 2013). Even in the planarian flatworm, a remarkable organism capable of regenerating any of its tissues after transection, primitive macrophage-like cells are abundant at the site of injury (Peiris et al., 2014). The conservation of this phenomenon across the animal kingdom gives further weight to the idea that remyelination requires a robust inflammatory response.

## How do innate immune cells contribute to remyelination?

**Figure 1:**
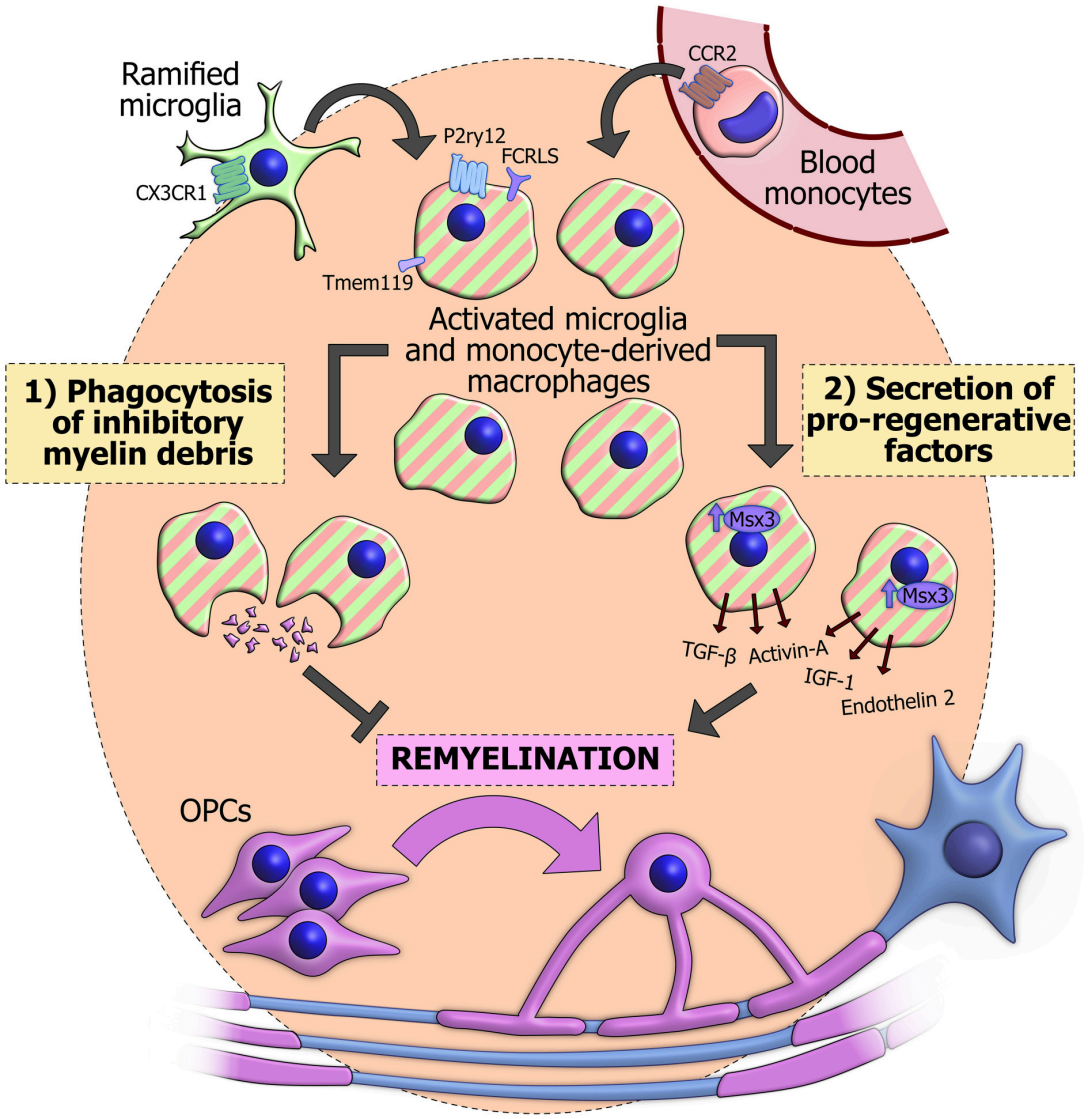
**The actions of innate immune cells during remyelination**. Innate immune cells in a demyelinated lesion can derive from activation of CX3CR1+ resident ramified microglia (green). Alternatively they may originate from blood monocytes (pink), which are recruited from the circulation in a CCR2-dependent manner and differentiate into macrophages. CX3CR1 and CCR2 have proved useful for genetically labeling or ablating the separate populations. Once activated, these cells become difficult to distinguish based on morphology and classical immunohistochemical techniques (striped), though some surface markers have recently been observed specifically in microglia, including Tmem119, P2ry12, and FCRLS. Innate immune cells can phagocytose inhibitory myelin debris and secrete an array of pro-regenerative factors, some of which are positively regulated by the transcription factor Msx3. The combination of these functions promotes the differentiation of OPCs (purple) and subsequent reinvestment of new myelin sheaths around denuded axons (blue).

The most important functions of innate immune cells in remyelination appear to be (1) phagocytosis of debris and (2) secretion of growth signals, cytokines and other factors. Through, a combination of these mechanisms, microglia, and monocyte-derived macrophages can fashion a pro-regenerative environment that maximizes the potential of OPCs to differentiate and replace the myelin on denuded axons (Figure 1).

Macrophages are well known for their phagocytosis of invading pathogens, cellular debris and apoptotic host cells. In a lesion where myelin sheaths are destroyed, much of this phagocytic response is directed toward fragments of myelin, which can linger in the environment. CNS myelin inhibits OPC differentiation *in vitro* (Robinson and Miller, 1999) and *in vivo* it was shown that remyelination is impaired if a lesion is supplemented with additional myelin debris (Kotter et al., 2006). This would suggest that clearance of myelin debris by phagocytosis is necessary for OPCs to produce new oligodendrocytes. More direct evidence for this comes from recent studies showing reduced remyelination when phagocytosis is specifically impaired in microglia (CX3CR1 knockout, Lampron et al., 2015) or in infiltrating macrophages (LysM-specific RXRα knockout, Natrajan et al., 2015).

Inflammation can also contribute to remyelination through phagocytosis-independent mechanisms. This is apparent during the enhanced myelination of retinal ganglion cells by transplanted OPCs when inflammation is stimulated (Setzu et al., 2006). These axons are not myelinated under normal conditions, so there is accordingly no myelin debris for innate immune cells to clear. Innate immune cells can secrete a wide array of factors that contribute to the lesion environment, and these are disturbed in older animals, which have a lower capacity for remyelination (Hinks and Franklin, 2000; Zhao et al., 2006). The ability of microglia-conditioned media to modulate OPC behavior *in vitro* is further evidence of secreted factors promoting remyelination. Media conditioned by pro-inflammatory M1 microglia, which appear soon after a lesion, promote OPC proliferation, whilst media conditioned by M2 anti-inflammatory microglia, which peak later, prevent apoptosis and encourage differentiation to oligodendrocytes (Miron et al., 2013).

Several specific factors are known to be produced during inflammation and promote remyelination. Insulin-like growth factor (IGF-1) and transforming growth factor-β (TGF-β) have long been known to promote OPC differentiation *in vitro* (McMorris and Dubois-Dalcq, 1988; McKinnon et al., 1993) and their expression is delayed in the slow remyelination of old rats (Hinks and Franklin, 2000). More recently, endothelin 2 was identified by transcriptional profiling of the OPC retinal transplant model (Yuen et al., 2013) and activin-A was found to be essential for the microglia-conditioned media effects on OPC differentiation (Miron et al., 2013). The msh-like homeobox-3 gene (*Msx3*) appears to be an important positive regulator for the M2 phenotype and for expression of activin-A and IGF-1. Overexpression of *Msx3* in transplanted microglia enhances remyelination in both EAE and lysolecithin models (Yu et al., 2015).

## Divergent roles for macrophages and microglia

The two key functions of phagocytosis and mediator secretion can be carried out by both resident microglia and infiltrating macrophages, which are often discussed as a single functional population. However, as our models of remyelination and the tools to probe them become more sophisticated, more differences between these two pools of innate immune cells are emerging.

A comparison of the transcriptomes of microglia and peripherally-derived macrophages revealed several hundred genes that could distinguish between the two populations, largely related to sensing endogenous ligands and microbes (Hickman et al., 2013). This may become less pronounced when microglia become activated in a lesion environment, as inflammatory stimuli can substantially alter the microglial transcriptome (Bodea et al., 2014). Nevertheless, it is possible to determine the origin of innate immune cells in EAE lesions based on maintained molecular signatures: notably, the microglial-specific surface markers P2ry12, FCRLS (Butovsky et al., 2014), and Tmem119 (Bennett et al., 2016) are maintained during inflammation, and are not upregulated in monocyte-derived macrophages. Prior to these recent advances, the use of blood-brain barrier impermeable toxins have been used to specifically ablate peripherally-derived macrophages, whilst fluorescent bone-marrow chimeras have allowed peripheral cells to be tracked upon entry to the CNS.

These techniques have demonstrated how microglia and infiltrating macrophages have a high degree of overlap in their function. Microglia appear to undergo a compensatory proliferation in lesions lacking monocyte-derived macrophages due to peripheral ablation (Kotter et al., 2005). Using a green fluorescent protein-positive (GFP^+^) bone marrow chimera, it was shown that the transition from M1 to M2 phenotypes occurs similarly in both microglia and infiltrating macrophages after a lysolecithin-induced lesion (Miron et al., 2013). The infiltration of young monocytes into an old lesion in the heterochronic parabiosis model accelerates the shift from M1 dominance to M2 dominance within the lesion and likely contributes to the enhanced remyelination. Relatively few cells in the lesion were derived from a GFP^+^ young partner, suggesting that these infiltrating cells were influencing endogenous innate immune cells to create a pro-regenerative environment.

Other experiments have elucidated divergent functions of microglia and monocyte-derived macrophages in remyelination. When demyelination was induced by dietary cuprizone, blocking peripheral monocyte infiltration by CCR2 deficiency was inconsequential to the regenerative process (Lampron et al., 2015), in contrast to the importance of this process for efficient remyelinating of a sterotactic lysolecithin lesion (Ruckh et al., 2012). Endogenous microglia, on the other hand, were shown to be vital as remyelination was impaired in a CX3CR1 microglial receptor knockout mouse model. In the dietary cuprizone model, demyelination occurs without the blood-brain barrier damage of stereotactic injection (Bakker and Ludwin, 1987), which may account for the reduced role of monocyte-derived macrophages, though these cells were still able to infiltrate the lesioned CNS (Lampron et al., 2015). Additionally this study did not look at aging, which may be a context in which CCR2-dependent monocyte recruitment becomes more critical. Divergent roles for endogenous and infiltrating components were also observed when EAE was induced in transgenic mice with green CX3CR2+ microglia and red CCR2+ macrophages (Yamasaki et al., 2014). In this case, highly activated monocyte-derived macrophages appear to initiate demyelination, whilst microglia had more beneficial roles in clearing debris.

This rapidly expanding body of evidence is leading us away from a model of a single macrophage/microglia compartment in a demyelinated lesion. Significant transcriptional differences in sensome genes (Hickman et al., 2013) suggest that the two populations can respond differently to the same environmental stimuli. The manifestation of these differences seems to depend strongly on the experimental model, perhaps implying that differences in age or blood-brain barrier function are important (Ruckh et al., 2012; Lampron et al., 2015). In EAE, where the pathology is immune-mediated, infiltrating macrophages and resident microglia may even have opposing roles, promoting de- and re-myelination respectively (Yamasaki et al., 2014). However, this result contrasts with a beneficial effect of specifically blocking resident microglial activation, even in EAE with the same immunogen (Heppner et al., 2005). In any case, the high variability between experimental setups suggests that the relative contributions of these two innate immune cell populations will likely differ between individual clinical myelin disorders, their stages and lesion types.

## Inflammatory therapies for regeneration in myelin disease?

A range of clinical diseases involve primary demyelination, the causes of which can be intrinsic or extrinsic to the oligodendrocyte lineage. Intrinsic causes account for the leukodystrophies, in which genetic mutations affect production of myelin proteins and other oligodendrocyte functions. Alternatively, the pathology can originate from the oligodendrocytes' environment, for example inflammation in MS, or toxicity as in many model systems. Whilst cell therapy is a promising avenue for leukodystrophies, diseases that originate externally will most likely benefit from interventions that lessen the demyelinating insult and promote endogenous remyelination (Franklin and Goldman, 2015).

Currently, most disease-modifying drugs used to treat MS focus on modulating the adaptive immune response using small molecules or monoclonal antibodies. These can be effective in reducing the length and frequency of relapses and provide symptomatic relief, though there is limited impact on the progressive phase of the disease and no curative agents are currently in clinical use (Goldenberg, 2012). Modulating the innate immune response to promote endogenous remyelination is a potential means to salvage denuded axons and prevent progression of the disease.

Microglia and infiltrating macrophages promote remyelination through important roles in debris clearance and secretion of factors into their local environment. These two functions become gradually less efficient as an animal ages (Hinks and Franklin, 2000; Ruckh et al., 2012; Natrajan et al., 2015), though aspects of the decline appear to be reversible by altering the tissue environment (Linehan et al., 2014). Compelling evidence from parabiosis shows that remyelination by previously inefficient OPCs in an old animal can indeed be rejuvenated by manipulating components of the innate immune system (Ruckh et al., 2012). Systemic pharmacological activation of the innate immune system has also been shown to promote remyelination (Döring et al., 2015). Additionally, as we learn more about the differences between microglia and monocyte-derived macrophages, divergent roles may be specifically targeted to enhance the production of a pro-regenerative environment.

However, the immune system is a complex network with much cross-talk between its adaptive and innate branches. Translational challenges will come in being able to stimulate the beneficial functions of innate immune cells without simultaneously fuelling further autoimmune destruction of myelin sheaths. Additionally, many of the symptoms of MS result from the death of axons that have already been too long without the trophic support and protection of myelin (Franklin and Gallo, 2014). Endogenous remyelination could not reverse the symptoms associated with previous axonal death, though it could salvage vulnerable axons at the point between demyelination and death. As such, remyelinating therapies would address an important unmet need in the treatment of demyelinating disease. Such regenerative therapies will likely be additive and complementary to the current disease-modifying treatments that can reduce the occurrence of demyelination with variable risks of adverse effects.

Despite foreseeable challenges, harnessing the pro-regenerative properties of inflammation has shown extensive benefit in animal models of remyelination. This approach has the potential to partially reverse the course of MS when it begins to makes the transition from bench to bedside.

## Author contributions

All authors listed, have made substantial, direct and intellectual contribution to the work, and approved it for publication.

## Funding

The authors would particularly like to acknowledge the support of the UK MS Society, The Jean Shanks Foundation, and MedImmune. Research in RJMF's laboratory is supported by a core support grant from the Wellcome Trust and MRC to the Wellcome Trust-Medical Research Council Cambridge Stem Cell Institute.

### Conflict of interest statement

The authors declare that the research was conducted in the absence of any commercial or financial relationships that could be construed as a potential conflict of interest.
